# Contribution of microvascular dysfunction to chronic pain

**DOI:** 10.3389/fpain.2023.1111559

**Published:** 2023-02-02

**Authors:** Terence J. Coderre

**Affiliations:** Department of Anesthesia and Alan Edwards Centre for Research on Pain, McGill University, Montreal, QC, Canada

**Keywords:** endothelial cells, microvascular injury, ischemia-reperfusion, oxidative stress, antioxidants, cytokines

## Abstract

There is growing evidence that microvascular dysfunction is a pathology accompanying various injuries and conditions that produce chronic pain and may represent a significant contributing factor. Dysfunction that occurs within each component of the microvasculature, including arterioles, capillaries and venules impacts the health of surrounding tissue and produces pathology that can both initiate pain and influence pain sensitivity. This mini review will discuss evidence for a critical role of microvascular dysfunction or injury in pathologies that contribute to chronic pain conditions such as complex regional pain syndrome (CRPS) and fibromyalgia.

## Introduction

While pain physicians and researchers have for centuries concentrated on the role of nerve and inflammatory injury to chronic pain and have more recently studied the potential role of neuron-glia interactions, very little attention has been paid to microvascular function. This is surprising given that a healthy microvasculature is critical for endoneurial health, and microvascular injury plays a significant role in various components of the inflammatory response to tissue damage.

Endoneurial microvessels are essential to the control of ion, solute and water transfer between the bloodstream and the endoneurium. These functions allow for nutrient, macromolecule and leukocyte influx and efflux, as well as contributing to interstitial fluid balance between layers of the endoneurium (1). Alterations in these processes may result in nerve damage that leads to neuropathic pain.

**Figure 1:**
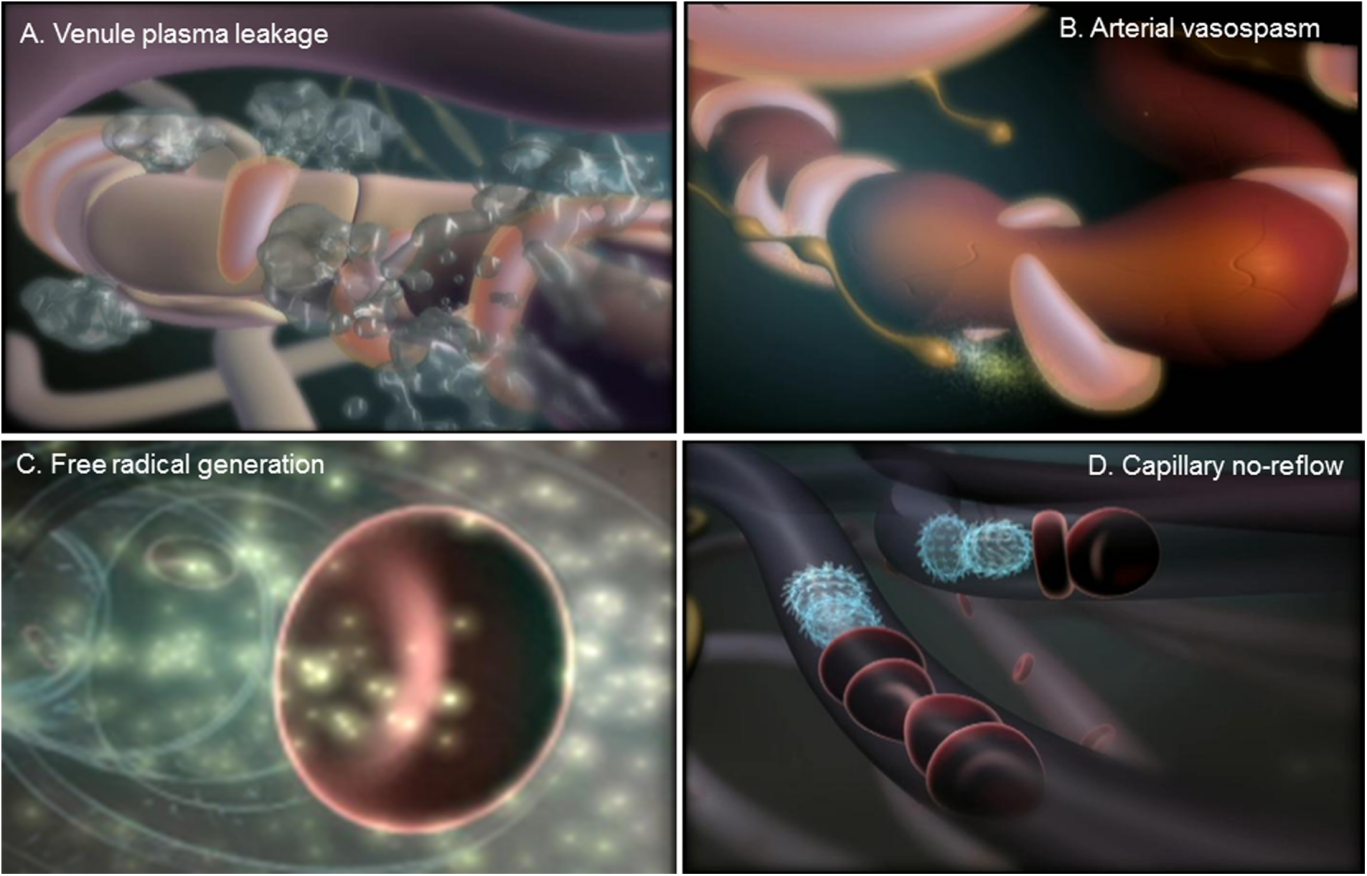
Microvascular response to tissue injury. (**A**) Swelling or edema is caused by a leakage of plasma proteins from microvessels within the post-capillary venules. (**B**) Extensive edema within damaged tissue can cause vasospasms within arterioles that can produce prolonged tissue ischemia. (**C**) Reperfusion of microvessels in the ischemic tissue generates the production of oxygen and nitrogen free radicals, which produce injury of the endothelial lining, attraction and adhesion of platelets and leukocytes, activation of the coagulation cascade, and increased thrombosis. (**D**) The adhesion of platelets and leukocytes leads to a blockade of blood flow within capillaries, or the phenomenon capillary no-reflow, which encourages arteriovenous shunting and the enhanced proliferation of blood and lymphatic vessels.

All three element of the microvasculature contribute to inflammation (see Figure 1), with arterioles involved in impaired vasomotor function, capillaries critical for reduced perfusion and poor oxygenation of tissue, and post-capillary venules playing a role in increased vascular permeability (2). These actions trigger various other inflammatory responses including the adhesion of platelets and leukocytes, activation of the coagulation cascade, increased thrombosis, and the enhanced proliferation of blood and lymphatic vessels (3).

In the current minireview, I will assess the role of microvascular dysfunction in two chronic pain conditions, complex regional pain syndrome (CRPS) and fibromyalgia. I concentrate on these two syndromes due to both the elusive nature of their etiologies and/or to the growing evidence of the role played by microvascular dysfunction to their underlying pathologies. A brief section will also discuss the role of the microvasculature in other chronic pain syndromes, such as migraine and neuropathic pain.

### Evidence for microvascular dysfunction in CRPS

CRPS, until recently known as reflex sympathetic dystrophy, is a chronic pain condition that usually follows deep-tissue injuries such as fractures, crush injuries or sprains, with incidence estimates as high as 26.2 per 100,000 person years (4). Symptoms of CRPS include spontaneous pain (”burning” pain referred to the skin, and “aching” pain referred to deep tissues), and a variety of stimulus-evoked abnormal pain sensations, including mechanical allodynia and hyperalgesia, cold allodynia, and sometimes heat hyperalgesia (5). CRPS-I pain is disproportionate to the injury and is not associated with injury of a major nerve, unlike CRPS-II a similar syndrome, but with concomitant major nerve damage. Non-painful symptoms of CRPS include disorders of vasomotor and sudomotor regulation; trophic changes in skin, hair, nails, and bone; and dystonia and other motor abnormalities (5). Vascular abnormalities are prominent symptoms in CRPS patients, with either increased or decreased skin temperature observed, typically corresponding with increased or decreased blood flow (6, 7), and alterations in bone (8, 9). However, it is a common finding that CRPS vascular symptoms change over time. The general picture often reported is a short initial “hot” phase lasting weeks to months, followed by a “mixed hot/cold” phase, and finally a chronic “cold” phase lasting for years (10, 11). During the “hot” phase, the limb displays increased skin temperature, edema, sweating and inflammation, while in the “cold” phase it shows lowered skin temperature, dryness, and cyanosis.

There is a growing body of evidence which suggests that at least some cases of CRPS may also depend on ischemia in deep tissues, and occasionally skin, secondary to limb trauma. Muscle tissue obtained after amputation of the affected limbs of 8 CRPS patients was found to exhibit lipofuscin pigment, atrophic fibers, and collapsed lumens and severely thickened basal membrane layers of the capillaries (12). These findings are consistent with oxidative stress, lipid peroxidation and ischemic conditions resulting from microvascular injury in deep tissue. Although amputation may reflect the most severe cases of CRPS, this conclusion is supported by observations of increased density of perfused vessels; lower capillary filtration capacity, and arteriovenous shunting of peripheral subcutaneous tissue in the intact limbs of patients with CRPS (13, 14). Co-incident with these changes appears to be high arterial flow to the CRPS limb, but low oxygen consumption (indicated by high venous oxygen saturation), as well as high lactate flux—indicative of tissue ischemia despite increased flow in large vessels (15, 16). There is also an impairment of high-energy phosphate metabolism in muscle tissue of CRPS limbs (16, 17), suggestive of mitochondrial oxygen deficiency. A role of oxidative stress in CRPS is suggested by finding that symptoms are relieved in these patients following treatment with antioxidants and free radical scavengers (18, 19). These observations suggest that ischemia in deep tissues may contribute to the induction and maintenance of CRPS. Increased activity in the sympathetic vasoconstrictor innervation of the affected limb would exacerbate any underlying ischemic condition and might be an important contributor to ischemia in some cases. It has also been shown that skin capillary hemoglobin oxygenation (HbO_2_) or skin nutritive flow is reduced in CRPS patients (6, 10, 20) limbs. CRPS patients can have either hot limbs with high blood flow, or cold limbs with reduced blood flow, but whether patients experience pain or not, is independent of the direction of the alteration in skin temperature or thermoregulatory flow (10, 21).

The findings summarized above indicating that there may be microvascular dysfunction and ischemia in deep tissues of CRPS patients, suggest that a disruption of nutritive flow may be a more important factor underlying pain in CRPS patients than changes in thermoregulatory flow. Thus, although thermoregulatory flow may be low or high, reduced nutritive flow to deep tissue in either case would reduce oxygenation leading to tissue ischemia.

### Evidence for microvascular dysfunction in fibromyalgia

Fibromyalgia (FM) is a chronic pain condition that affects about 2%–3% of the adult population. The defining feature of FM is chronic widespread pain, but patients often exhibit other symptoms, including fatigue, sleep disturbance, irritable bowel syndrome, mood disorders and headache (22, 23). Although the underlying causes of FM are not well understood, the syndrome has been associated with medical illness, stress, various other pain conditions, as well as disturbances in various neurotransmitter and neuroendocrine systems (23, 24). There is also evidence for both peripheral and central sensitization in FM, as well as dysfunction of descending inhibitory control systems (24, 25). Widespread pain is one of the diagnostic criteria for FM, with the common diagnostic requirement of tenderness in 11 of 18 defined tender points (26). Pain is described as deep, gnawing or burning, and can be accompanied by stiffness, skin tenderness, pain after exercise, paraesthesias, restless legs, and Raynaud's phenomenon, a process in which the fingers or toes become blanched in response to cold or stress (27). Theories about the role of muscle pathology in FM have been controversial (28), although numerous studies have reported histological abnormalities in muscle tissue (29, 30).

The controversy over the role of muscle pathology in FM likely stems from findings that the muscle pathology is subtle, and typically detected by histological rather than functional assessments (28). Studies indicate that muscle pathology includes capillary dysfunction and abnormalities of mitochondria in myocytes (31–33). Mitochondrial abnormalities are significant since mitochondria provide the energy source in the form of ATP through oxidative phosphorylation, and function poorly when not supplied with enough oxygen (34). Recent studies do indicate that there are signs of oxidative stress or increases in oxygen free radicals in FM patients. Thus, in FM patients there is increased serum malondialdehyde and lipid hydroperoxide, indicators of free radical-induced lipid peroxidation, and decreases in xanthine oxidase, which is normally depleted when oxygen free radicals are produced (35, 36). There are also decreases in the endogenous antioxidants – glutathione and superoxide dismutase and total antioxidant status in serum, which would be depleted in response to oxidative stress (35, 37). FM patients also have higher levels of oxygen free radicals in blood mononuclear cells (38), and neutrophils from FM patients produce greater levels of the oxygen free radical hydrogen peroxide (39). This may explain why antioxidants have been useful as analgesics for fibromyalgia in some studies (40).

Additional evidence suggests that oxidative stress in FM patients may occur in response to microvascular dysfunction in affected muscle tissue and possibility in skin. Indeed, it has been shown that FM patients have reduced capillary flow and fewer capillaries in the nail fold (31, 32), perhaps explaining why Raynaud's phenomena is common in FM patients (41). As for muscle, recent microdialysis studies suggests that FM patients have reduced nutritive blood flow and increased lactate in skeletal muscle in response to exercise (42). These results support earlier findings of reduced capillaries, thickened capillary endothelial cells and lowered ATP in biopsies from the vastus lateralis muscle of FM patients (33). These are consistent with findings of reduced muscle blood flow in FM patients as measured using xenon 133 clearance (43), and reduced muscle blood flow in cervical spine trapezius of FM patients following exercise (44). There was also one report indicating that muscle tissue oxygenation is lower in the tender point areas of FM patients (45). Also, while blood flow and skin temperature has been reported to be either lower (46) or higher (47) over tender points in FM patients, there is no correlation between skin temperature and pressure pain threshold (48).

### Evidence for microvascular dysfunction in other pain syndromes

The space limitations of mini reviews prevent a detailed description of similar pathology within in other pain syndromes. However, there is considerable evidence for a role of microvascular dysfunction contributing to chronic pain in various syndromes such as angina, frostbite, sickle cell disease, and peripheral vascular disease (49, 50). Data suggest a role for dysfunction in the endoneurial tissues of patients with painful diabetic neuropathy (51, 52), and experimental animals with neuropathic pain (53). There is also evidence that patients with migraine have contributing or associated alterations in microvascular function within the retina (54), dura matter (55), cerebral cortex (56) and coronary tissue (57).

## Conclusion

In this mini review, we summarized the potential contribution of microvascular dysfunction to the chronic pain conditions CRPS and fibromyalgia. Evidence of microvascular dysfunction is exhibited by numerous findings of thickened capillary endothelial cells, disruptions in capillary or nutritive blood flow, increases in lactate and oxidative stress, and arteriovenous shunting and abnormalities in mitochondria for both conditions. Numerous studies have concentrated on the effects of mediators released from various immune cells, including mast cells, neutrophils, macrophages and T-cells and their impact on neural function. Far fewer studies have investigated the role of mediators released by or from endothelial cells, including adhesion factors, endothelins, cytokines, chemokines, selectins, additional vasodilatory and vasoconstrictive factors, antithrombotic and procoagulant factors, matrix products, endothelial growth factors, and others, on both immune and neural function (58). It is hoped that future research and targeted therapy will be aimed at microvascular dysfunction and its role in the etiology and maintenance of symptoms in these and potential other chronic pain syndromes. These studies would add to the above-mentioned studies demonstrating the effectiveness of antioxidants or free radical scavengers as analgesics for CRPS (18, 19) and fibromyalgia (40), and why combinations of topical agents aimed at improving both arterial and capillary blood flow (59), or salts and co-crystals comprised of vasodilators and antioxidants are effective analgesics in animal models of CRPS and neuropathic pain (60).
